# The transverse and longitudinal elastic constants of pulp fibers in paper sheets

**DOI:** 10.1038/s41598-021-01515-9

**Published:** 2021-11-17

**Authors:** Caterina Czibula, August Brandberg, Megan J. Cordill, Aleksandar Matković, Oleksandr Glushko, Chiara Czibula, Artem Kulachenko, Christian Teichert, Ulrich Hirn

**Affiliations:** 1 Institute of Physics, Montanuniversitaet Leoben, Franz Josef Str. 18, 8700 Leoben, Austria; 2 Institute of Bioproducts and Paper Technology, Graz University of Technology, Inffeldgasse 23, 8010 Graz, Austria; 3 Christian Doppler Laboratory for Fiber Swelling and Paper Performance, Graz University of Technology, Inffeldgasse 23, 8010 Graz, Austria; 4 Solid Mechanics, Department of Engineering Mechanics, KTH Royal Institute of Technology, Teknikringen 8D, 114 28 Stockholm, Sweden; 5 Erich Schmid Institute for Materials Science, Austrian Academy of Sciences, Jahnstr. 12, 8700 Leoben, Austria; 6 Department Materials Science, Montanuniversitaet Leoben, Jahnstr. 12, 8700 Leoben, Austria

**Keywords:** Biomaterials, Materials science

## Abstract

Cellulose fibers are a major industrial input, but due to their irregular shape and anisotropic material response, accurate material characterization is difficult. Single fiber tensile testing is the most popular way to estimate the material properties of individual fibers. However, such tests can only be performed along the axis of the fiber and are associated with problems of enforcing restraints. Alternative indirect approaches, such as micro-mechanical modeling, can help but yield results that are not fully decoupled from the model assumptions. Here, we compare these methods with nanoindentation as a method to extract elastic material constants of the individual fibers. We show that both the longitudinal and the transverse elastic modulus can be determined, additionally enabling the measurement of fiber properties in-situ inside a sheet of paper such that the entire industrial process history is captured. The obtained longitudinal modulus is comparable to traditional methods for larger indents but with a strongly increased scatter as the size of the indentation is decreased further.

## Introduction

Paper and paperboard are made from mechanically or chemically processed wood fibers mixed with water and sprayed onto a moving mesh. Using gravity, heat, and pressure, the water is forced out, which enables the fiber surfaces to bond with each other to form a sheet. At the macroscopic scale, the main predictors of mechanical performance are the sheet density and the production process, such as the pulping method, the amount and type of chemical additives, and the drying conditions (cf. Chapter 13 of Ref.^1^). However, the separation of length scales is weak, and the network structure, fiber orientation, fiber mechanical properties, and ability of fibers to bond affect the macroscopic mechanical response^1^. The mechanical properties of the individual fibers—the main building blocks of paper and cellulose-based composites—are, therefore, of interest in several situations, most importantly in modeling and product optimization efforts.

**Figure 1:**
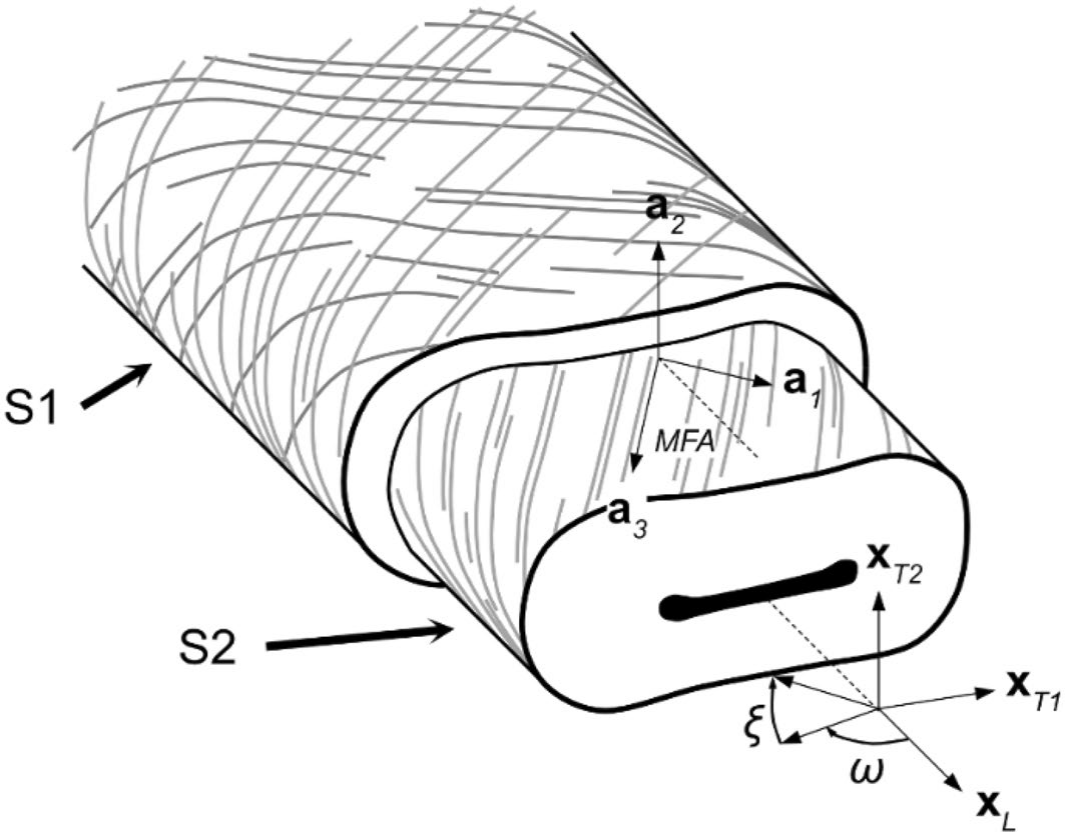
Schematic illustration of a pulp fiber. Most of the fiber volume is in the S2 layer, while the S1 layer forms an outer shell. During the papermaking process, the S1 layer is damaged, partially exposing the underlying S2 layer. The fiber is characterized by the base $\{{{\varvec{x}}}_{T1}, {{\varvec{x}}}_{T2},{{\varvec{x}}}_{L}\}$, while the material base is represented by $\{{{\varvec{a}}}_{1},{{\varvec{a}}}_{2},{{\varvec{a}}}_{3}\}$. Relative to the fiber axis, the microfibrils are aligned at an angle, typically between 0° and 40°^1^, illustrated here by the angle between the axis ${{\varvec{x}}}_{L}$ and ${{\varvec{a}}}_{3}$. The S2 layer is transversely isotropic, with the axis of symmetry (the strong direction) oriented along ${{\varvec{a}}}_{3}$. The S1 layer has a less well-defined fibril orientation, which is, here, represented by two different orientations of the fibrils in the plane of the fiber surface.

Wood fibers have a hierarchical structure, as shown schematically in Fig. 1^1^. The fiber wall is composed of several layers, each layer made up of cellulose, lignin, and hemicellulose^1^. The cellulose is organized in long, mostly parallel chains called fibrils. The orientation of these fibrils is characterized by the microfibril angle (MFA), measured between the longitudinal axis of the fiber and the longitudinal axis of the fibrils.

Testing individual pulp fibers is difficult due to the small and non-uniform dimensions of the fibers (length of 1–5 mm, width of 10–30 µm, thickness of 3–15 µm). The fiber is anisotropic with a stiffer response along the axis of the fibrils. The material response is usually described using a transversely isotropic model^1^. Since the axial properties are the most important for the sheet properties and the easiest to measure experimentally, most efforts have concentrated on determining the longitudinal fiber elastic modulus, strength, and strain to failure. The mechanical properties in other directions have been less investigated (cf. Chapter 14 in Ref.^1^). Characterization of individual fibers can be done by isolating them, fixing them in place, and subjecting them to load using a uniaxial tensile testing machine. Although much work has been presented on the longitudinal mechanical properties of individual fibers^2,3^, serious challenges in isolating, manipulating, and restraining the sample during the test still exist. The fiber is usually not straight when unloaded, and the microfibril angle introduces an axial–torsional coupling that causes restraints on the cross-section’s rotation to stiffen the axial response (cf. p. 757–764 in Ref.^1^). Furthermore, most of the literature have focused on the uniaxial tensile testing of single fibers isolated prior to sheet forming or of wood fibers that were never pulped^2,3^. However, micromechanical properties should ideally be probed after subjecting the material to the real process history to ensure that the properties are representative of those in the finished product. Wuu showed that the elastic modulus of fibers surgically extracted from sheets shrinking freely during drying was less than half of that when fibers were dried individually^4^. Kappil et al. found that in sheets dried under restraint in one direction and freely in the other, there is a correlation between the mechanical properties of the fiber and its orientation relative to the direction in which shrinkage was prevented^5^.

To avoid testing individual fibers in this way, an option is to use a model relating the material properties of the fibers to the material properties of the sheet. This is a convenient approach, as testing sheets is straightforward. Many such so-called network models have been proposed, beginning with Cox’ theory^6–8^. There are two drawbacks of this approach. First, network models usually express the sheet modulus as a function of fiber characteristics. This requires the expression to be inverted to obtain the material properties of the fiber. Most network models cannot guarantee the uniqueness of the modulus if obtained in this way, as they are not closed-form expressions or cannot be analytically inverted. Second, almost all such models rely on physically motivated but experimentally unavailable parameters that, in practice, function as fitting parameters. This raises questions of how transferable the parameter estimates are outside the assumptions of the chosen network model.

Nanoindentation (NI) is another alternative to uniaxial tensile testing of individual fibers. This approach has been used to investigate the wall of spruce wood tracheids^9–12^ as well as pulp fibers^13,14^ that were isolated prior to sheet forming. The most common result from indentation tests is the indentation modulus (sometimes called the reduced modulus), a property describing the elastic response of the indented body. However, interpretation of this quantity is less straightforward than in uniaxial testing because indentation of anisotropic materials yields a response that is a mix of stiffness tensor components. Vlassak et al. and Swadener et al. presented models to predict the indentation modulus in the elastic anisotropic case^15,16^. Jäger et al. adapted Vlassak’s model to study wood fibers and employed an inverse modeling scheme to obtain estimates of the longitudinal, transverse, and shear modulus from indentations along different normals^17,18^.

NI can be employed to study the properties of fibers inside the sheet after production if the sheet is cut using a microtome to expose the fibers in the bulk of the sheet. This allows direct access to the longitudinal cross-section of the fiber. An obstacle to this approach is that conventional NI machines tend to have limited resolution in the range of low loads suitable when studying pulp fibers. Applying higher loads creates larger indents and introduces questions of how the boundary of the fiber influences the obtained mechanical response. This is one reason why NI has not been applied much to paper and paperboard. For smaller loads, atomic force microscopy (AFM)-based NI methods have become a standard technique and can be used to apply loads in the pN‒µN range. The small size of the indenter helps overcome the high surface roughness of the fiber surface^19–21^.

In this work, we apply NI and AFM-NI to pulp fibers in the bulk of paper sheets that were exposed via microtome cutting and compare the results to longitudinal single fiber tensile testing data, as well as to the results obtained using a network model. We show that NI methods provide estimates of the longitudinal elastic modulus similar to those obtained by micromechanical testing while also yielding an additional material parameter. The sources of uncertainty in NI experiments are largely different from those in micromechanical testing and network models.

## Results

### Morphological and chemical analysis

**Figure 2:**
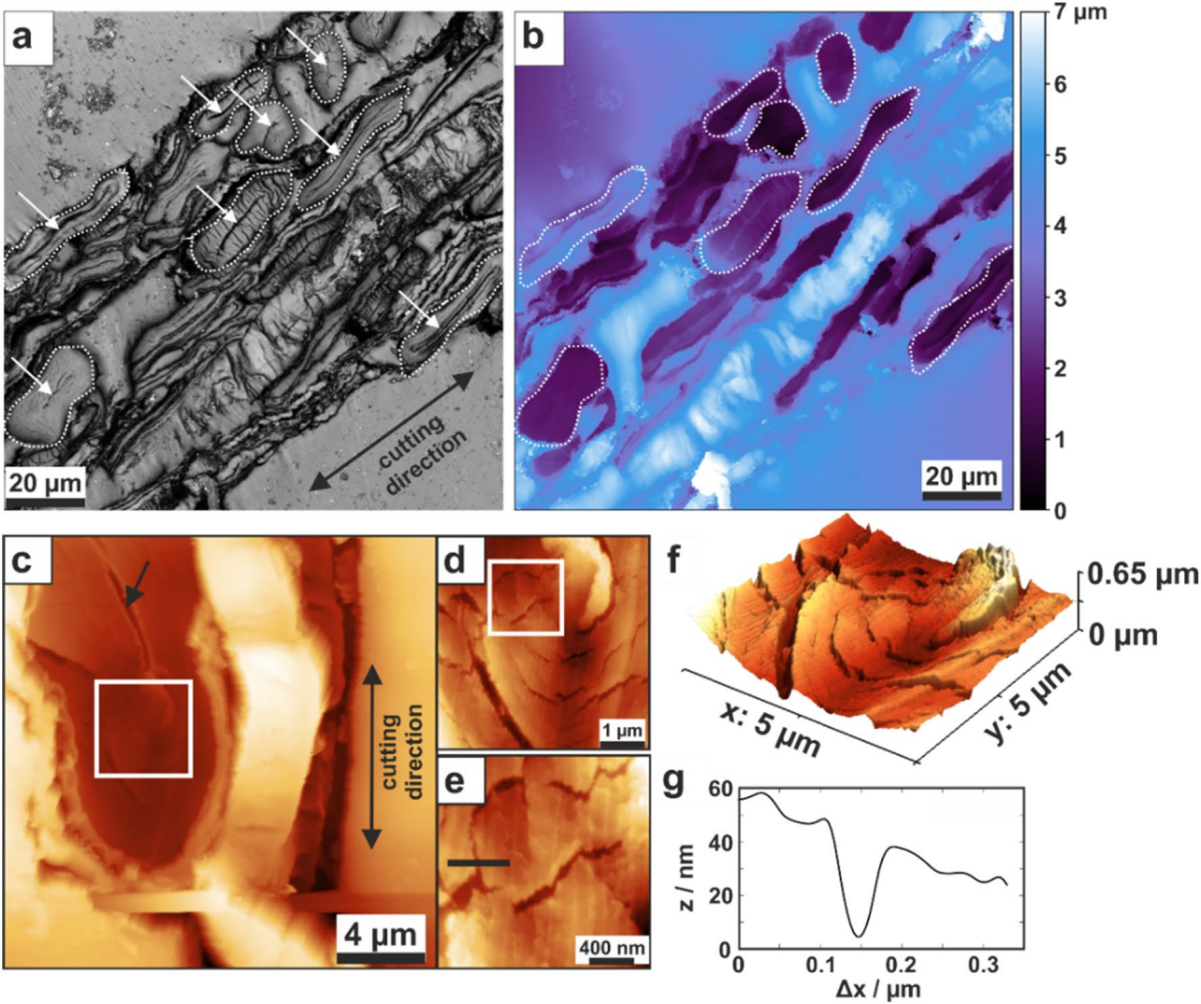
(**a**, **b**) Confocal laser scanning microscopy (CLSM) images of the surface of a microtome-cut slice of paper. (**a**) Laser intensity image and (**b**) corresponding height image. The white contours mark fiber cross-sections suitable for indentation. Each arrow in (**a**) indicates a collapsed lumen of a fiber. (**c**–**f**) AFM topography images. (**c**) 20 × 20 μm^2^ topography image of a fiber cross-section cut perpendicular to the longitudinal fiber axis (z-scale: 4 μm). The black arrow points at the collapsed lumen, and the white squares indicate the zoom-in regions. Zoom-ins: (**d**) 5 × 5 μm^2^ topography image (z-scale: 650 nm) and (**e**) 1 × 1 μm^2^ topography image (z-scale: 300 nm). The black line indicates a cross-section to determine the width and depth of a crack, which is illustrated in (**g**). (**f**) 3D representation of the topography image in (**d**). (**g**) Cross-section of a crack indicated by the black line in (**e**).

The microtome-cut fiber surface was investigated using confocal laser scanning microscopy (CLSM) to obtain large overview images. In Fig. 2, a CLSM intensity image with the corresponding height map is presented. Most of the identifiable fibers have been cut perpendicular to the longitudinal fiber direction. Fibers that have been cut parallel to the longitudinal direction are not easily distinguishable. In Fig. 2a, most of the visible fiber cross-sections exhibit a collapsed lumen, indicated by white arrows. Fibers that were deemed suitable for mechanical characterization are marked by white dashed contours. The height image in Fig. 2b indicates that the embedding material usually exceeds the surface of the fiber cross-sections. Topography images obtained by AFM are presented in Fig. 2c–f. In Fig. 2c, the surface of a fiber cross-section is presented with high resolution details in Fig. 2d–f. The measurement of line-scans, as presented in Fig. 2g, indicates a crack width of about 100 nm and a depth of about 50 nm. For the indentation routines, care was taken to avoid regions close to these cracks.

**Figure 3:**
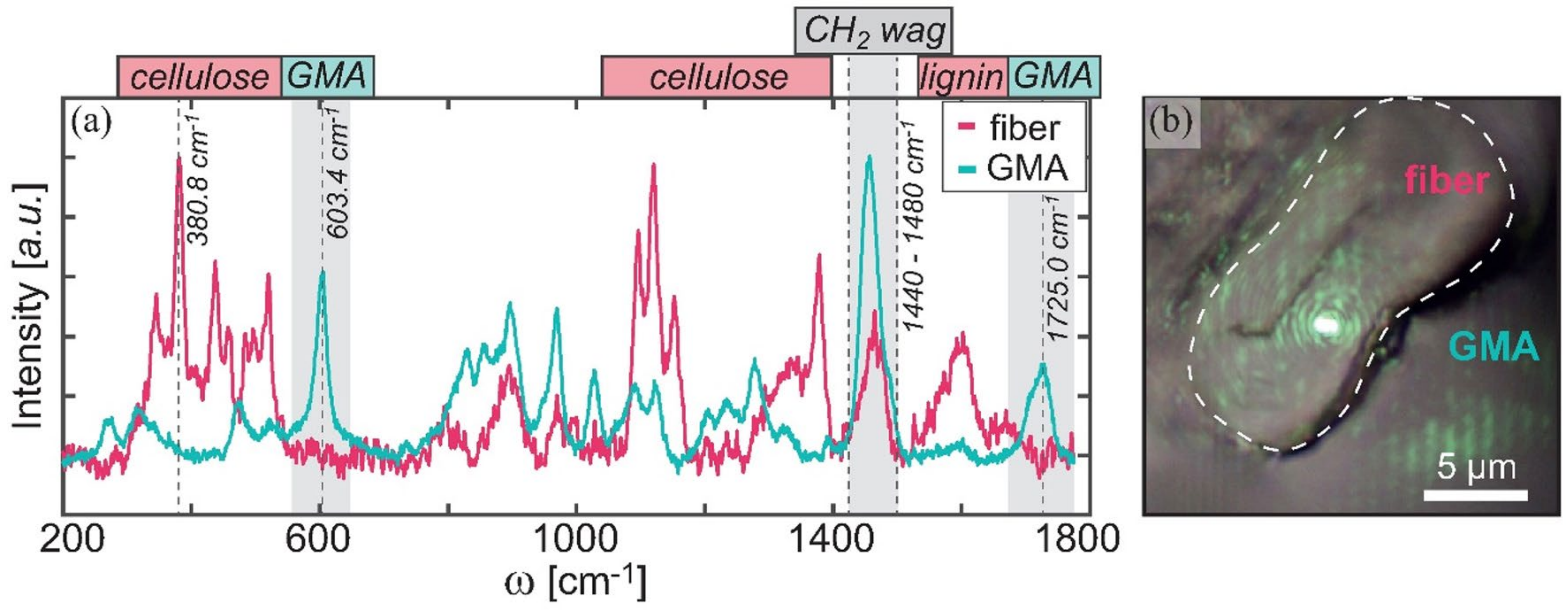
(**a**) Raman spectra of the fiber and the surrounding GMA. Two characteristic modes of GMA (at 603.4 cm^−1^ and 1725 cm^−1^) that were not observed in the fibers are highlighted. CH_2_ wagging mode (1440–1480 cm^−1^ range) and a characteristic cellulose mode at 380.8 cm^−1^ are also highlighted. (**b**) 20 × 20 µm^2^ optical microscopy image of the fiber embedded in GMA. The dashed white line marks the fiber perimeter. The laser spot on the sample is visible and marks the spot from which the Raman spectrum (**a**) of the fiber was measured. For the reported GMA spectrum, the laser spot was positioned in the bottom-right of (**b**).

A comparison between the Raman spectra of the fiber and the glycol methacrylate (GMA) is presented in Fig. 3a. The measurement positions are indicated in Fig. 3b. In the spectrum of the fiber, mainly characteristic cellulose modes (in the ranges of 300–500 cm^−1^ and 1100–1400 cm^−1^), a lignin characteristic mode at about 1600 cm^−1^, and a CH_2_-wagging mode (within the 1440–1480 cm^−1^ range) are visible^22^. In the case of GMA, the CH_2_-wagging mode is also observed, and two additional modes at 603.4 cm^−1^ and at 1725 cm^−1^, which are characteristic of GMA, are detected^23^. Since these two modes are not visible in the spectra measured on the fibers, we assume that embedding the fibers in GMA to produce microtome slices has a negligible influence on the mechanical properties of the fibers during the indentation experiments.

### Mechanical characterization

The Cox network model (Eq. 1) can be inverted to obtain the longitudinal fiber modulus $E_{L}^{{\text{(fiber)}}}$. The modulus of the sheet and the density of the sheet are given in Table 1 together with the estimate of the fiber density. The fiber modulus derived in this way is 15.2 GPa. The main uncertainty is the density of the fiber, where the value used assumes that the fiber has no voids.

**Table 1:** Mechanical properties of tested handsheets and fiber density relevant to the Cox equation (Eq. 1).

$E^{{({\text{sheet}})}}$	1.55 ± 0.07	GPa	This study, n = 24
$\rho^{{({\text{sheet}})}}$	459 ± 10.0	kg m^−3^	This study, n = 6
$\rho^{{({\text{fiber}})}}$	1500	kg m^−3^	Assumed based on p. 788 in Ref.^1^

Measured properties are given as mean ± standard deviation.

**Figure 4:**
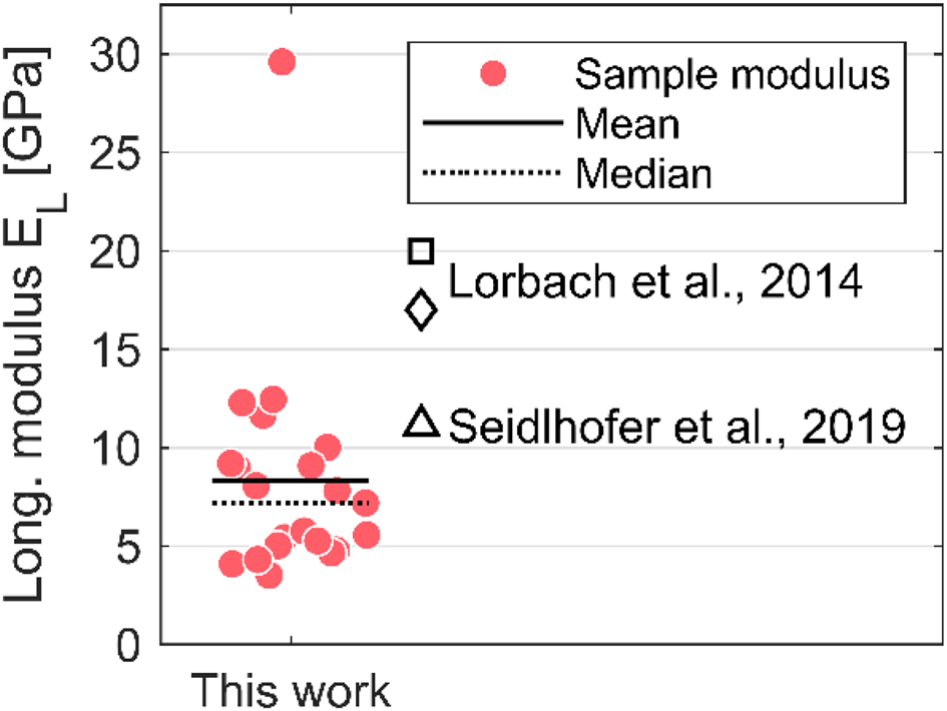
Longitudinal moduli obtained from micromechanical tensile tests in comparison to three mean estimates from previous studies on the same pulp^24,25^. The experimental results are spread on the x-axis to improve visibility.

In Fig. 4, the longitudinal elastic moduli $E_{L}$ obtained by micromechanical testing of individual pulp fibers and evaluation using Eq. (2) are presented as well as three mean estimates reported on the same pulp in previous works^24,25^. The longitudinal modulus ranges from 3.5 to 29.6 GPa, and the distribution of responses is skewed towards lower values. The mean (median) modulus is 8.3 (7.2) GPa.

**Figure 5:**
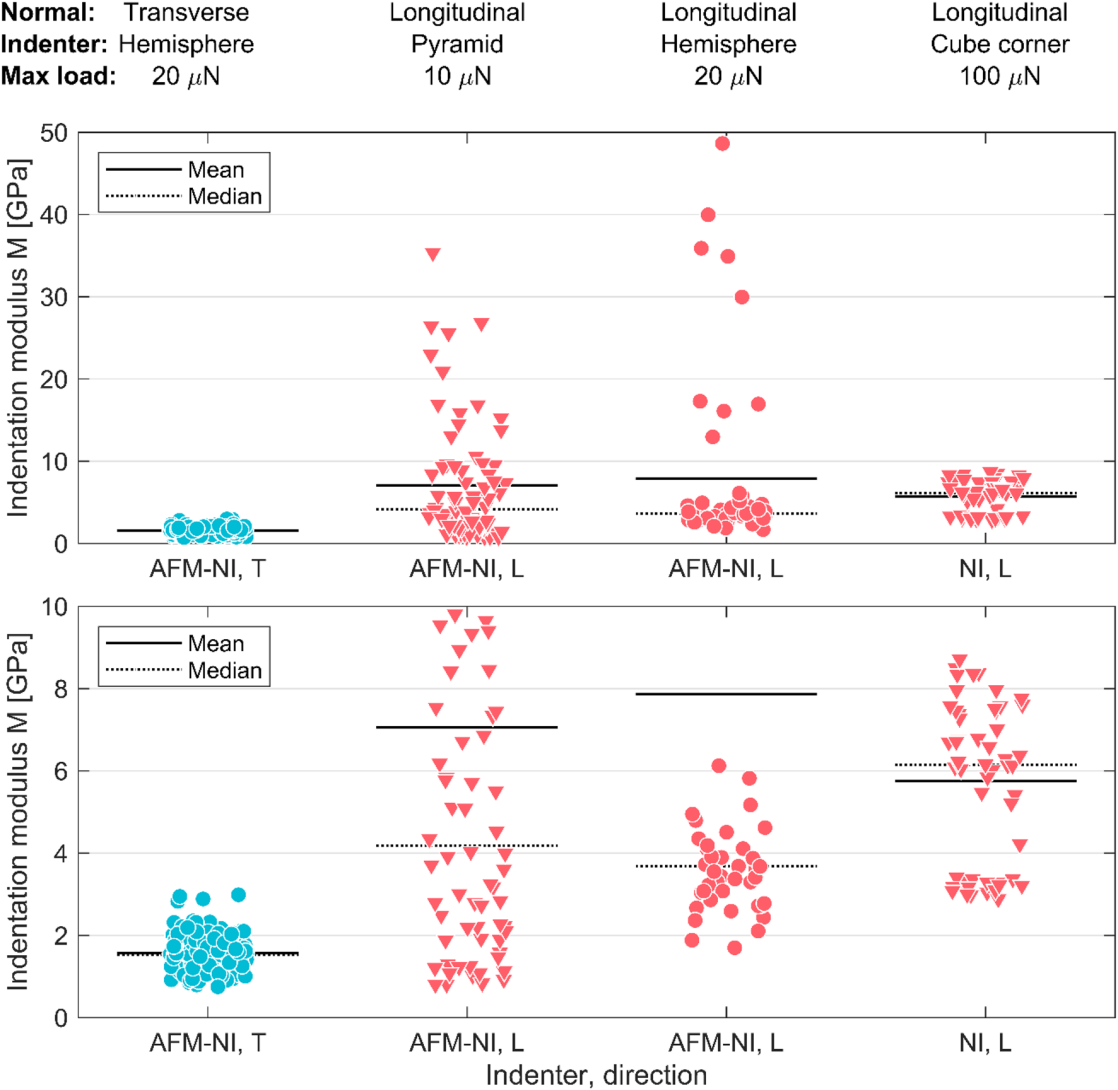
Indentation moduli obtained by AFM-NI and NI in the transverse (T) and longitudinal (L) direction with pyramidal/cube corner (▼) and hemispherical (●) indenters. The lower subplot is a zoomed in version of the upper subplot.

Figure 5 shows the indentation moduli ${M}_{L}$ and ${M}_{T}$ measured with pyramid- and hemispherically shaped tips by AFM-NI and a cube corner indenter by conventional NI. The transverse indentation modulus ${M}_{T}$ obtained by AFM-NI is clearly lower than the ${M}_{L}$ values of AFM-NI and NI. The longitudinal indentations exhibit scatter, especially for the AFM-NI indentations. Both indenter shapes yield similar results for ${M}_{L}$. The NI results, where a larger testing force of 100 µN is applied, exhibit less variance and a higher median but a lower mean response compared to the AFM-NI results. The AFM-NI data from the longitudinal direction show a clear skew, while the values for the transverse direction and the NI indentations form more symmetrical distributions.

Due to the skew in the indentation data distribution, it is relevant to consider both the mean and the median indentation modulus. The median value is less sensitive to outliers, which is clearly important given the skew in the distribution of the indentation moduli. However, the mean value is expected to be the most representative of the indentation modulus if the entire cross-section could be probed simultaneously.

Using literature values for the Poisson’s ratio $\nu_{TT}$^26^ and the shear modulus $G_{TL}$^27^, which are given in Table 2, Eq. (15) was applied to estimate the longitudinal and transverse elastic modulus $E_{L}$ and $E_{T}$ of the fibers. For AFM-NI with a pyramidal (hemispherical) indenter, taking the mean indentation modulus resulted in a longitudinal elastic modulus $E_{L}$ of 11.1 GPa (13.3 GPa) and a transverse elastic modulus $E_{T}$ of 1.1 GPa (1.1 GPa), while using the median indentation modulus yielded *E*_*L*_ = 4.7 GPa (3.8 GPa) and *E*_*T*_ = 1.1 GPa (1.2 GPa). The difference between the estimates stems directly from the difference in the indentation moduli in the longitudinal direction. The longitudinal indentations using NI combined with the AFM-NI transverse indentations were less sensitive to whether the mean or the median was used, giving an estimate of *E*_*L*_ = 8.0 GPa, *E*_*T*_ = 1.1 GPa (means) and *E*_*L*_ = 8.9 GPa, *E*_*T*_ = 1.1 GPa (medians).

**Table 2:** Inputs for the determination of the unknown parameters ${E}_{L}$ and ${E}_{T}$ based on measurements with AFM-NI and NI and literature data obtained from the same type of pulp.

Material property		Conditions	Source
${{\varvec{M}}}_{{\varvec{L}}}$		45% relative humidity, 25 °C	This study
AFM-NI hemisphere	Mean: 8.57 GPa		
	Median: 3.67 GPa		
AFM-NI pyramid	Mean: 7.06 GPa		
	Median: 4.19 GPa		
NI	Mean: 5.75 GPa		
	Median: 6.15 GPa		
${{\varvec{M}}}_{{\varvec{T}}}$		45% relative humidity, 25 °C	This study
AFM-NI hemisphere	Mean: 1.57 GPa		
	Median: 1.54 GPa		
${\nu }_{TL}$	0.25		Assumed
${\nu }_{TT}$	0.25	50% relative humidity, 23 °C	^26^
${G}_{TL}$	2.51 GPa	50% relative humidity, 23 °C	^27^
$MFA \,\, \theta$	0		Assumed

**Figure 6:**
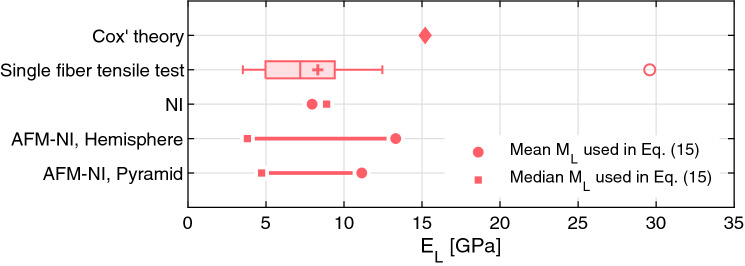
Estimates of the longitudinal modulus ${E}_{L}$ of the fibers from nanoindentation experiments (AFM-NI and NI) compared to the ${E}_{L}$ values obtained by single fiber tensile testing and Cox’ theory. The box plot for the single fiber tensile test consists of median (line in box), mean (cross in box), interquartile range IQR (box), whisker length 1.5·IQR, and outliers (open circles).

In Fig. 6, the estimates from this estimation routine are compared with the previously discussed moduli obtained using Cox’ theory and tensile testing. It is important to note that (1) the moduli used in Eq. (15) are averages, and (2) that the specific fibers tested with each method are not the same specimens. The nanoindentation results match those from the tensile testing. The Cox theory estimate is somewhat higher, and the AFM-NI results show a strong influence on the averaging scheme used to obtain the representative moduli.

In the Electronic Supplementary Materials (ESI), Sect. S5, the uniqueness of the obtained moduli pair output by Eq. (15) is investigated by performing a grid search over the realistic ranges of longitudinal and transverse moduli. There is a single cost function minimum. This holds true for all the other combinations used in Fig. 6. The transverse modulus predicted using Eq. (15) is 1.1 GPa. This estimate showed little variation when changing the value of the indentation modulus in the longitudinal direction. It is important to note that due to the absence of an alternative method against which to compare the transverse modulus obtained in this way, the accuracy of this estimate cannot be directly evaluated.

## Discussion

The ranges of longitudinal moduli obtained by the three methods largely overlap. There is considerable scatter in both the indentation and the micromechanical testing data. This scatter is absent when using the Cox' theory. Part of this reduction in variance stems from the larger volume tested, averaging over all fibers in the tested network. However, in the Cox' theory, the uncertainty is not entirely removed but rather hidden in the estimate of the fiber density and the quality of the required assumptions. In this work, we were able to measure the sheet modulus and the density of the sheet, but the density of the fiber was taken from literature, and it is possible that it is too high.

In Table A1 in the ESI, previously reported results from AFM-NI and NI measurements are summarized. The indentation moduli obtained in this work are significantly lower than those reported elsewhere, also compared to pulp of similar origin and process history^14^. As shown in Fig. 6, this seems to be due to the material rather than the method since all obtained results were similar. The scatter in the indentation moduli in the longitudinal direction is significant when applying AFM-NI, whereas the scatter is much lower in NI testing. It is known that the fiber cross-section is made up of distinct cellulose and non-cellulose phases with a large difference in stiffness^1^. Previous work on other multi-phase materials has demonstrated how this difference is smeared when using a large indenter but that different phases can be distinguished as the indentation size goes below the characteristic size of the phases^28^. The use of a nanoindentation approach could be further extended to study non-elastic response^29^, to decompose multi-phase response^28^ and adhesion properties (p. 147–196^30^), as well as to explore the humidity dependence of the fiber wall’s mechanical response. Introducing additional cutting planes would also allow the determination of the shear moduli, similar to what was done in Ref.^18^. An additional method of determining these parameters is desired, as only a few investigations have been presented so far.

## Conclusions

We tested three different methods of determining the longitudinal elastic modulus of the fibers: the Cox network model, single fiber tensile testing, and nanoindentation at two different scales. Each method requires several assumptions regarding the material behavior, and this is the first time these three methods were compared directly in this way.

Nanoindentation was used to characterize the fibers after they had been formed into a sheet, which has not been reported before. The indentation moduli obtained were combined with the model of indentation of transversely isotropic media proposed by Delafargue and Ulm^31^ to relate the observed indentation modulus to the stiffness tensor components. Three load levels and indenter geometries were used. The longitudinal elastic modulus obtained in this way ranges from 3.8 to 13.3 GPa, depending mostly on the postprocessing choice of whether to use the mean or the median as a representative estimate of the fiber response. When using a larger indenter, this discrepancy disappears, leading to a stable estimate of 8–9 GPa. This estimate compares well with the mean modulus obtained using single fiber tensile testing, (8.3 GPa). Furthermore, we note that the scatter in the tensile single fiber testing data (3.7–29.6 GPa) is large. Although the scatter in the longitudinal elastic modulus when using the AFM-NI is large, the use of AFM-NI is critical to obtain the transverse indentation modulus (1.1 GPa). Given the surface roughness and limited force resolution, accessing the transverse direction with NI, may not be possible without further sample preparation.

Compared to previous studies, the indentation modulus when indenting the longitudinal direction of the fiber was lower in this study. Single fiber testing corroborated the low modulus. In light of these findings, the elastic properties of the fibers should not be sourced from the literature when building a model for specific, predictive purposes.

Inverting the Cox network model to obtain an estimate of the longitudinal modulus resulted in a higher estimate (15.2 GPa) than testing individual fibers directly. Given the assumptions of the Cox model it would be natural to consider the Cox estimate as a lower bound for the fiber longitudinal elastic modulus. In fact, the estimate from Cox’ theory is higher than the direct tests, contrary to expectation. This demonstrates that material properties obtained in this way are not fully decoupled from the model assumptions.

In practice, it is not possible to answer the question of which of the tested methods is superior without knowledge of the intended use of the data. Still, some recommendations can be given.

- Directly testing the fibers is preferable to using a network model if the material property estimates are to be used in other models.
- Single fiber tensile testing and NI testing of the fibers yield approximately the same longitudinal modulus. Since the assumptions used in these two methods do not overlap, this provides some support for assuming that the surface roughness, boundary effect, and sample preparation do not significantly affect the estimate in nanoindentation testing.
- As the indentation size is further decreased, the indentation response in the longitudinal direction of the fiber exhibits a larger scatter. The source of this scatter, be it from the ultrastructure of the fiber cross-section or a method deficiency, is unknown and requires further investigation.

The success of the work, especially the comparison of NI and tensile testing of single fibers, suggests more advanced load schedules could be a way to extract more material data via indentation studies.

## Materials and methods

### Pulp and sheets

The material investigated is an industrially produced unbleached, unrefined, once-dried softwood Kraft pulp (Mondi Frantschach, Austria) made from a mixture of spruce and pine. The lignin content of the pulp was characterized by its kappa number *κ* = 42. The pulp was swollen in deionized water for 24 h, and then sheets were prepared according to ISO 5269-2 using a Rapid-Köthen handsheet former. A few handsheets were microtomed and used for indentation studies. In addition, six in-plane isotropic handsheets with a surface weight of 80.4 ± 1.4 g m^−2^ were cut into a total of 24 test specimens. After equilibration of the sheets in standard conditions according to ISO 554 (23 °C, 50% relative humidity (RH)) for at least 24 h, the in-plane elastic modulus of the sheet $E^{{\text{(sheet)}}}$ was determined by a tensile test with an L&W Tensile Tester (Lorentzen & Wettre, Germany) according to ISO 1924-2. To directly access the transverse direction of fibers inside the sheet, individual fibers were carefully separated from the spare sheets by a combination of peeling less dense layers from the sheet and separating fibers with tweezers to expose transverse surfaces from the bulk of the sheet. Microtome slices of the handsheets were prepared to enable access to the fiber cross-sections in the bulk of the sheet. For indentation along the axis of the fiber, the sheet was first embedded in a glycol methacrylate resin (GMA)^32,33^ and then cut using a diamond blade to a slice thickness of approximately 7 µm. Samples were attached to a steel sample holder using nail polish as previously described^19^.

### Raman spectroscopy

Raman analysis with a Labram HR Evolution spectrometer (Horiba, France) was used to confirm that the resin does not penetrate the fiber wall. A confocal configuration was employed, and Raman spectra were recorded with a 532 nm laser (50 mW power on the sample surface), a 600 l/mm grating, a 100× magnification lens, 6 s acquisition times, and 10 accumulations. The influence of cosmic rays was removed from the spectra, and intensities were normalized by the Labspec 6 software. To ensure that there was no laser-induced damage, multiple Raman spectra were measured from the same spots.

### Confocal laser scanning microscopy

A confocal laser scanning microscope (CLSM) LEXT 4100 OLS (Olympus, Japan) was used for optical 2D and 3D imaging of the fiber cross-section samples. The laser wavelength was 405 nm, and the lateral resolution was 120 nm. The CLSM images were analyzed by the Gwyddion software^34^.

### Micromechanical tensile tests

A dynamic mechanical analysis (DMA) device (DMA 850, TA Instruments, USA) was used to perform tensile testing of 20 single pulp fibers at 50% RH and 23 °C. The fibers were fixed to an acrylic glass sample holder with a span length of 0.85 ± 0.10 mm (mean ± standard deviation) by a two-component glue (UHU Endfest) according to the procedure described in^35^. Displacement-controlled tensile tests were performed with a preload of 5 mN to a maximum displacement of 15 µm with a rate of 10 µm/s. The cross-sectional area of each fiber was determined by embedding the fibers in resin and microtome-cutting 4–5 slices per fiber. Each slice was inspected by optical microscopy, and the cross-sectional area was estimated by applying a MATLAB routine to a binarized image of the cross-section. The average cross-sectional area of each fiber was used to estimate the longitudinal modulus of that fiber. The 20 fibers tested had an average cross-sectional area of 249 ± 96 µm﻿.

### Atomic force microscopy-based nanoindentation

AFM-based nanoindentation (AFM-NI) measurements were performed with an MFP-3D AFM (Asylum Research, USA) equipped with a closed-loop planar x–y-scanner with a scanning range of 85 × 85 µm^2^ and a z-range of about 15 µm. For topography imaging, AC160TS-R3 silicon probes (Olympus, Japan) with an aluminum reflective coating on the backside of the cantilever and a nominal tip radius of 10 nm were used. The AFM images were analyzed by the Gwyddion software^34^.

Two different probe geometries were selected for AFM-NI. First, ND-DYIRS probes (Advanced Diamond Technologies, USA) were utilized. These probes are full-diamond probes with a reflecting aluminum backside coating of the cantilever and with a four-sided pyramid indenter tip. The angles of the indenter tip are all 45°. The cantilever spring constant was 82.1 ± 17.7 N/m as determined by the thermal sweep method^36^. The thermal Q factor was 623 ± 54 and the resonance frequency was 378 ± 14 kHz (mean ± standard deviation calculated from four independent measurements). Second, nominally hemispherically shaped LRCH250 silicon probes (Team Nanotec, Germany) with a reflecting aluminum backside coating of the cantilever were used. The spring constant of the cantilever was 290.2 ± 51.3 N/m. The thermal Q factor was 778 ± 224, and the resonance frequency was 575 ± 2 kHz (mean ± standard deviation calculation from three independent measurements). The tip geometries were characterized using the dilation principle^37^ with an AFM calibration grid (NT-MDT, Russia) with sharp spikes. The tip radii were obtained by fitting the surface profile of the indenter obtained in this way with paraboloids. For the pyramidal and the hemispherical probes, the tip radii were found to be 100 nm (here, only the cap of the pyramid was considered) and 300 nm, respectively.

The AFM test chamber was humidity-controlled using a closed fluid cell (Asylum Research, USA) at 45% RH, and the temperature varied between 24 and 27 °C during the measurements^19,21,38^. During the AFM-NI experiments, the surface was first scanned in tapping mode with the imaging probe. The surface of the longitudinal fiber cross-section was smooth with a root mean square roughness (RMS) of about 15 nm (obtained from 5 × 5 µm^2^ topography scans). The transverse fiber surface was rougher with an RMS of about 150 nm (obtained from 5 × 5 µm^2^ topography scans), but care was taken to find flat regions suitable for indentation.

**Figure 7:**
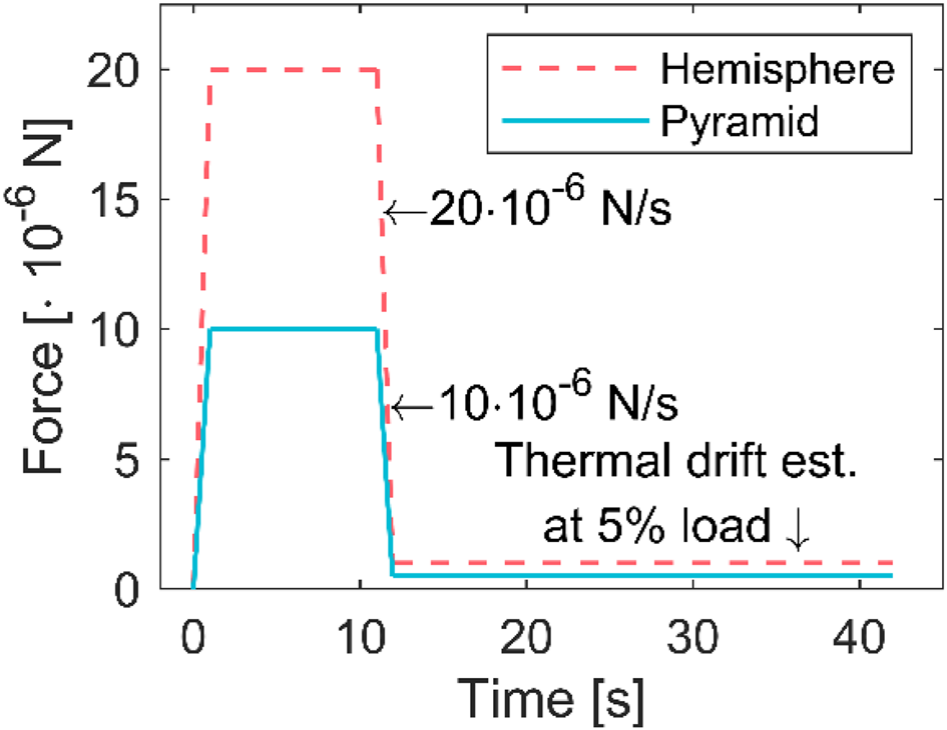
Load schedule of the AFM-NI experiments for the hemispherical and pyramidal probe.

The loading protocol is illustrated in Fig. 7 and consists of establishing the initial contact followed by a 1 s ramp to a force of 10 µN for the pyramid and 20 µN for the hemisphere. The load was held for 10 s followed by a force-controlled unload to 5% of the peak load, after which the thermal drift was estimated over 30 s. During the hold time, the force was actively controlled to remain at the target value. In the longitudinal direction, 50 indents (five individual fibers) and 54 indents (four individual fibers) were obtained with the hemispherical and pyramidal probe, respectively. In the transverse direction, the surface was indented with the hemispherical probe at 162 positions (three individual fibers). Typical curves and representative topography images of the sample surface before and after AFM-NI for both probes are shown in the ESI, Sect. S1.

### Nanoindentation (NI)

Nanoindentation was performed with a Bruker-Hysitron TS 77 Select (Bruker, USA) using a cube corner indenter. Appropriate indentation sites were identified by scanning the indenter across the prepared sample surface. Once suitable 10 × 10 µm^2^ areas were found by scanning in contact mode, indentation mapping was used to obtain 5 × 5 indents with a 1 µm spacing. This procedure was repeated for three individual fiber cross-sections and generated a total of 52 successful indents. The load profile was the same as the first part of the AFM-NI load schedule in Fig. 7 consisting of a 1 s ramp, 10 s hold, and 1 s unload in force-controlled mode. The Oliver and Pharr method^39^ was used to calculate the indentation modulus.

## Theory

### Sheet testing—Cox' theory

The longitudinal modulus of the fiber is related to the modulus of the sheet. Reducing the sheet to a collection of pin-jointed trusses and assuming the fibers are infinitely long yields Eq. (1), where $E_{L}^{{\text{(fiber)}}}$ is the longitudinal modulus of the fiber, $\rho^{(\textrm{sheet})}$ is the density of the sheet, and $\rho^{{\text{(fiber)}}}$ the density of the fiber^6^.

1 $$E^{{\text{(sheet)}}} = \frac{{E_{L}^{{\text{(fiber)}}} }}{3}\frac{{\rho^{{\text{(sheet)}}} }}{{\rho^{{\text{(fiber)}}} }}$$

### Longitudinal single fiber tensile testing

The longitudinal micromechanical single fiber testing is performed in the small strain regime (15 µm displacement imposed on a ~ 0.85 mm long fiber). A small preload eliminates any slack or non-straightness of the fiber. The load rate is high to prevent creep during the test. Under these conditions, the fiber is assumed to respond as an elastic truss. In that case, the cross-sectional area $A$, the applied force $F$, the applied displacement $\delta$, and the original fiber length $L$ can be used as in Eq. (2) to determine the elastic modulus of the fiber.

2 $$E_{L} = \frac{FL}{{\delta A}}$$

### Indentation analysis

**Figure 8:**
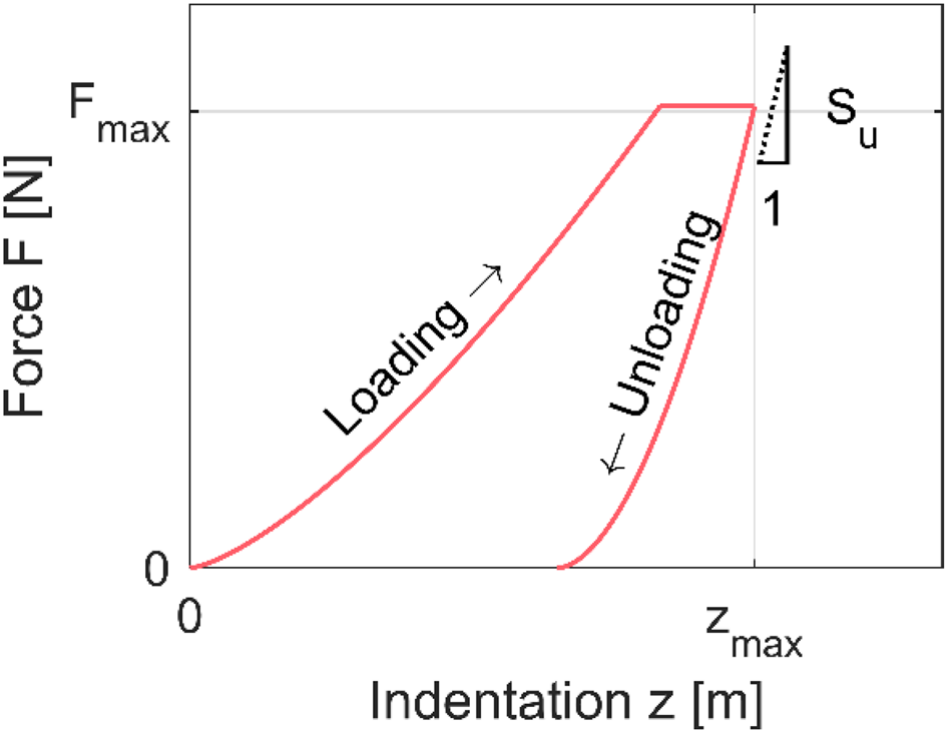
Force-Indentation depth plot illustrating the loading and unloading curve of the experiments. By determining the observed unloading slope S_u_, the indentation modulus M was calculated using Eq. (7).

The fiber is elastic, but some creep may appear during the indentation, as shown schematically in Fig. 8 where the displacement changes at constant peak load. This creep has been observed previously when testing wood and pulp^13^. This deformation is mostly a problem if no hold phase is introduced in the experiment. Under such conditions, the unloading stiffness may even become negative^40^. In addition to the use of a hold period at peak load, we compensate the creep at the beginning of unloading, as discussed below. The material properties are assumed to be transversely isotropic with the axis of symmetry oriented along the fiber axis. The fiber wall is a composite of stiff cellulose and compliant hemicellulose and lignin. The outer layers tend to be dissolved by the Kraft pulping process, and therefore, it is assumed that they influence the response only marginally.

The indentation force–displacement response looks schematically like in Fig. 8. The indentation modulus $M$ is obtained from the unloading path of the indentation experiments using the Bulychev–Alekhin–Shorshorov (BASh) relation given in Eq. (3), where $A(z_{c} )$ is the contact area and ${\text{d}}F/{\text{d}}z$ is the slope of the force–displacement curve^41^. $\beta$ is defined here as a correction for the fact that (1) the Galin–Sneddon indentation solution on which Eq. (3) is based neglects radial displacements of the half-space surface and assumes small-strain response and (2) the pyramidal indenter used here does not have a conical but rather a pyramidal cross-section. In other words, $\beta$ is a mix of the term $\Phi_{c}$ in^30^ and $\beta$ as defined in^29^. *β* = 1 for the hemispherical indenter and 1.05 for the pyramid indenter in this work, in line with best practices^29,42^.

3 $$M = \frac{1}{\beta } \cdot \frac{\sqrt \pi }{{2\sqrt {A(z_{c} )} }} \cdot \frac{{{\text{d}}F}}{{{\text{d}}z}}$$

The slope of the force–displacement curve during unloading and the contact area are determined using a modified version of the method proposed by Oliver and Pharr^39^. Many variations and improvements have been proposed to the method, e.g.^43,44^. Feng and Ngan noted that for material systems which exhibit creep, the functional form proposed by Oliver and Pharr (Eq. (14) in Ref.^39^) can result in a poor fit of the unloading slope close to the point of initial unloading, that is the critical part for the estimation of the indentation modulus^45^. To compensate for the effect of material creep during the load and hold phase of the experiment, the indenter-substrate system is idealized as a spring-dashpot system^45^ and the function given in Eq. (4) is used instead, where $z$ is the indentation depth, $D_{i}$ are fitting constants with appropriate dimensions, and $F$ is the indentation force.

4 $$z(F) \, = D_{1} + D_{2} \sqrt F + D_{3} F^{{D_{4} }}$$

Equation (4) is used to calculate the slope of the unloading by differentiating $z$ with respect to $F$ to obtain the unloading compliance $Q_{u}$ and then inverting to obtain the corresponding stiffness $S_{u}$, Eq. (5). Here, 75% of the unloading curve was used to fit Eq. (4). Machine compliance was compensated using the method suggested in Ref.^39^.

5 $$S_{u} = \frac{1}{{Q_{u} }} = \left( {\left. {\frac{{{\text{d}}z}}{{{\text{d}}F}}} \right|_{{F = F_{\max } }} } \right)^{ - 1}$$

The unloading slope is a mix of elastic unloading and continued creep of the material. Equation (6) is used to estimate the creep $z(t)$ where $t$ is the time measured from the onset of the hold phase and $D_{i}$ are fitting constants with appropriate dimensions.

6 $$z(t) = D_{5} + D_{6} t^{{D_{7} }}$$

Using Feng and Ngan’s method, the observed unloading stiffness $S_{u}$ is related to the elastic stiffness of the system $S$ via Eq. (7) where the displacement rate $\dot{z}(\tau_{u}^{ - } )$ is found by differentiation of Eq. (6) and where the unloading rate $\dot{F}$ is known. At the transition between the hold phase and the unloading phase at time $t = \tau_{u}$, the creep rate $\dot{z}(\tau_{u} )$ must be continuous^45^.

7 $$\frac{1}{S} = \frac{1}{{S_{u} }} - \frac{{\dot{z}(\tau_{u} )}}{{\dot{F}}}$$

The contact depth at unloading is determined via Eq. (8)^39^, adjusted for the effect of creep in the same way^46^. Here, $\varepsilon$ is a characteristic geometric constant to account for the fact that the indentation depth is less than the contact depth (cf. p. 1–27^30^). *ε* = 0.75 for hemispherical indenters and approximately 0.72 for conical indenters (p. 323–371^30^). Here, *ε* = 0.75 was used.

8 $$z_{c} = z_{\max } - \varepsilon \frac{{F_{\max } }}{S}$$

In anisotropic materials such as the one considered here, the indentation modulus obtained using Eq. (3) represents a mixture of the material stiffness components. It follows that the indentation modulus depends on how the indentation normal is oriented with respect to the material base. We introduce this dependence by two angles $\omega , \, \xi$ representing the azimuth and elevation with respect to the longitudinal axis of the fiber as shown in Fig. 1 to make this dependence explicit, $M = M(\omega ,\xi )$.

### Relating the indentation modulus to the stiffness tensor components

The indentation modulus represents the response of a combined elastic system with two components: the elastic response of the half-space and the elastic response of the indenter. These three sets of moduli (indentation, half-space, and indenter) are related via Equation (9) where $\psi ({\mathbb{C}}_{ijkm} ,\omega ,\xi )$ is a function describing the net contribution of stiffness tensor components ${\mathbb{C}}_{ijkm}$ for the indentation normal defined by the pair $\omega , \, \xi$, and $\nu_{i} , \, E_{i}$ are the Poisson’s ratio and Young’s modulus of the indenter, respectively.

9 $$\frac{1}{M(\omega ,\xi )} = \psi ({\mathbb{C}}_{ijkm} ,\omega ,\xi ) + \frac{{1 - \nu_{i}^{2} }}{{E_{i} }}$$

The indenter is significantly stiffer than the half-space, and therefore, the compliance of this component is disregarded. Equation (9) is derived under the assumption of frictionless interaction between smooth surfaces with small deformations compared to the radius of the indenter tip and the bodies in contact. We assume that these conditions hold here.

In this work, nanoindentation experiments were performed along the axis of the fiber as well as perpendicular to the fiber. If the MFA is assumed to be small and the fiber material is assumed to be transversely isotropic, the method by Delafargue and Ulm can be used to obtain explicit expressions for the indentation moduli as a function of the elastic stiffness components, as shown in Eqs. (10) and (11) for the longitudinal and transverse case, respectively^31^.

10 $$M(0,0) = M_{L} = 2\sqrt {\frac{{C_{1111} C_{3333} - C_{1133}^{2} }}{{C_{1111} }}\left( {\frac{1}{{C_{2323} }} + \frac{2}{{\sqrt {C_{1111} C_{3333} } + C_{1133} }}} \right)^{ - 1} }$$

11 $$M_{T} = \frac{1}{{2E(e)H_{2}^{3/4} H_{3}^{1/4} }}$$

Here, the help variables $H_{2} , \, H_{3}$ are given by Eqs. (12) and (13), while $e$ is the degree of ellipticity of the contact area (Eq. 14) and $E( \cdot )$ is the complete elliptic integral of the second kind of $\cdot$.

12 $$H_{2} = \frac{1}{2\pi }\sqrt {\frac{{C_{3333} }}{{C_{3333} C_{1111} - C_{1133}^{2} }}\left( {\frac{1}{{C_{2323} }} + \frac{2}{{\sqrt {C_{3333} C_{1111} } + C_{1133} }}} \right)}$$

13 $$H_{3} = \frac{1}{\pi } \cdot \frac{{C_{1111} }}{{C_{1111}^{2} - C_{1122}^{2} }}$$

14 $$e = \left\{ {\begin{array}{*{20}l} {\sqrt {1 - \frac{{H_{2} }}{{H_{3} }}} } \hfill & {{\text{if}}\;H_{2} < H_{3} } \hfill \\ {\sqrt {1 - \frac{{H_{3} }}{{H_{2} }}} } \hfill & {{\text{otherwise}}} \hfill \\ \end{array} } \right.$$

The method was presented for conical indenters, but comparison with the more general model proposed by Vlassak et al. reveals only marginal differences if a sphere is used instead^31,47^. This comparison is presented in the Electronic Supplementary Information (ESI). The effect of neglecting the MFA is also investigated (Sect. S3) and is shown to be small if the MFA is not significantly larger than 0 (< 20°).

Equations (10) and (11) define a relationship between the indentation moduli and the elastic stiffness components but are not analytically invertible. An inverse method is used to minimize the residual error between the measured indentation moduli $M^{\exp }$ and the predicted indentation moduli $M^{{{\text{model}}}}$, using the BFGS Quasi-Newton minimization scheme and the cost function $g(E_{L} ,E_{T} |\nu_{LT} ,\nu_{TT} ,G_{LT} ,\omega_{r} ,\xi_{r} )$ presented in Eq. (15), where $n$ is the number of indentation directions relative to the axis of the fiber. The other material parameters, $\nu_{LT} ,\nu_{TT} ,G_{LT}$, need to be sourced from other experiments unless additional cutting angles are introduced. Tensor manipulations were done using the MMTensor toolbox^48^.

15 $$g(E_{L} ,E_{T} |\nu_{LT} ,\nu_{TT} ,G_{LT} ,\omega_{r} ,\xi_{r} ) = \frac{1}{n}\sqrt {\sum\limits_{r = 1}^{n} {\frac{{(M^{{{\text{model}}}} - M^{\exp } )^{2} }}{{M^{\exp } }}} }$$

## Supplementary Information

Supplementary Information.

## Data Availability

The raw data required to reproduce these findings are available to download from 10.5281/zenodo.5163181^49^. The processed data required to reproduce these findings are available to download from 10.5281/zenodo.5163181^49^.

## Code availability

The code required to reproduce these findings is available to download from 10.5281/zenodo.5163181^49^.
