# Supplementary material for: The transverse and longitudinal elastic constants of pulp fibers in paper sheets
Source: Sci Rep. 2021 Nov 17;11:22411. doi: 10.1038/s41598-021-01515-9 (PMC8599457; doi:10.1038/s41598-021-01515-9)
Supplement: Supplementary file 1 — Supplementary Information. [file 41598_2021_1515_MOESM1_ESM.docx]

**Electronic Supplementary Information (ESI) for**

**The transverse and longitudinal elastic constants of pulp fibers in paper sheets**

**a**

Caterina Czibula, August Brandberg^*^, Megan J. Cordill, Aleksandar Matković, Oleksandr Glushko, Chiara Czibula, Artem Kulachenko, Christian Teichert, Ulrich Hirn

**S1: Experimental AFM-NI curves**


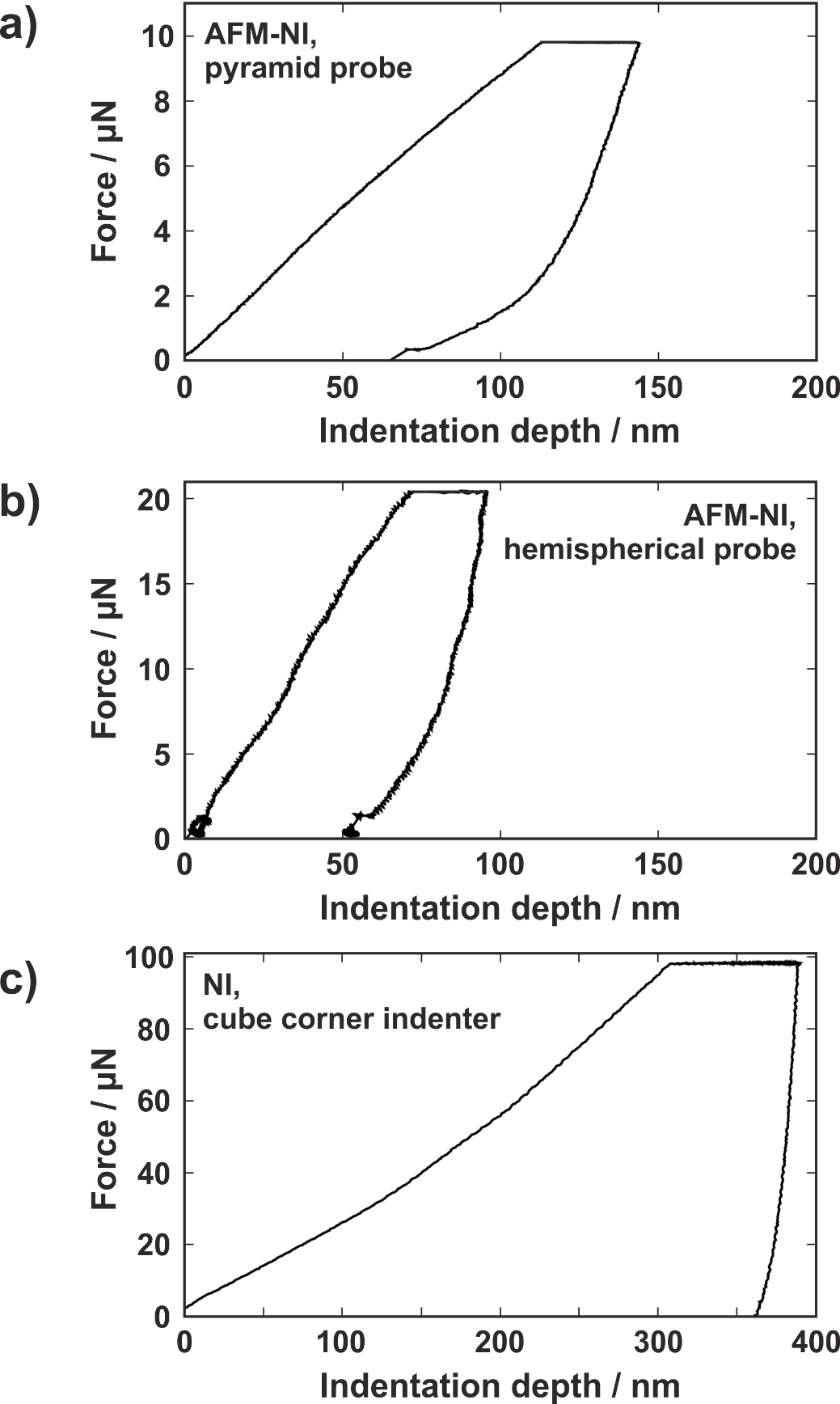


*Figure A 1: Representative force-indentation depth plots of the nanoindentation experiments for (a) AFM-NI with the hemispherical probe, (b) AFM-NI with the pyramidal probe, and (c) NI with a cube corner indenter.*


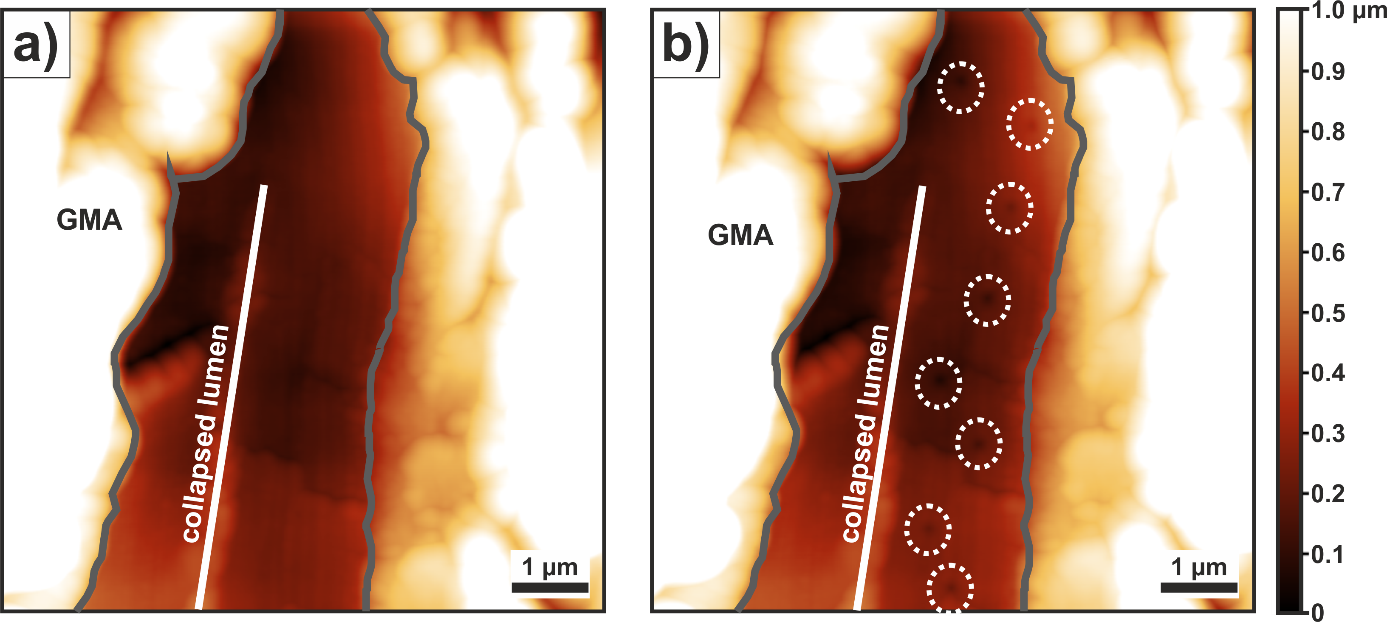


*Figure A 2: 8 x 8 µm² AFM topography images of a fiber cross-section (a) before and (b) after the nanoindentation experiment with the hemispherical probe. The black line encircles the fiber cross-section which is surrounded by the embedding resin (GMA) and the white straight line indicates the collapsed lumen. After the experiments, permanent indents from the plastic deformation are visible, which are marked with white dashed circles.*


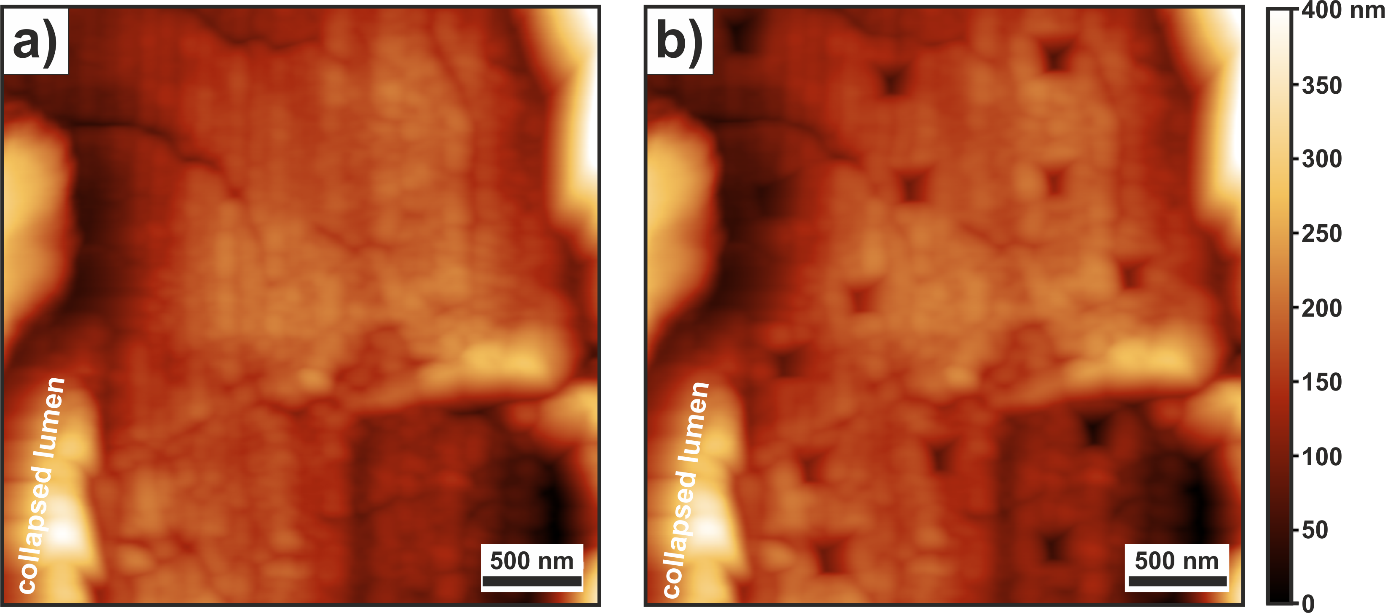


*Figure A 3: 3 x 3 µm² AFM topography images of a zoomed-in region on a fiber cross-section (a) before and (b) after the nanoindentation experiment with the pyramid probe. After the experiments, permanent indents from the plastic deformation are clearly visible.*

**S2: Overview of literature results for the S2 layer**

*Table A 1: Overview of the literature values of conventional nanoindentation (NI) measured on the S2 layer of wood and pulp fibers. Mean values obtained by AFM-NI at 45 % RH with two different probe geometries in this work are highlighted in blue for comparison.*

| ***wood species*** | ***detailed information*** | ***MFA / °*** | ***pulping grade*** | ***probe*** | ***E_r_ / GPa*** | ***H / MPa*** | ***Ref.*** |
| --- | --- | --- | --- | --- | --- | --- | --- |
| spruce | earlywood (EW) | x | x | Berkovich | 13.49 ± 5.75 | 254 ± 69 | [1] |
|  | transition wood (TW) | x | x |  | 21.27 ± 3.12 | 286 ± 39 |  |
|  | latewood (LW) | x | x |  | 21.00 ± 3.34 | 335 ± 30 |  |
| spruce | compression wood (CW) | 50 | x | Berkovich | 8.2 | ~450 | [2] |
|  | EW/LW | 0 | x |  | 17.1 | ~450 |  |
| spruce | LW | 3 | x | Berkovich | 15.81 ± 1.61 | x | [3] |
|  | LW | 5 | x |  | 15.34 ± 0.4 |  |  |
|  | LW | 5 | x |  | 17.08 ± 0.55 |  |  |
|  | TW | 7.5 | x |  | 13.46 ± 0.30 |  |  |
|  | TW | 7 | x |  | 17.54 ± 0.37 |  |  |
| eucalyptus | pulp | x | Unbleached (κ=13) | cube corner | 12.2 ± 1.6 | 420 ± 50 | [4] |
|  |  | x | Bleached (κ=1.5) |  | 10.5 ± 2.1 | 377 ± 33 |  |
| pine |  | x | Unbleached (κ=29) |  | 9.1 ± 1.6 | 430 ± 50 |  |
|  |  | x | Bleached (κ<1) |  | 10.5 ± 2.1 | 377 ± 33 |  |
| spruce/  pine | pulp | x | unbleached (κ<10) | Berkovich | ~16 | ~420 | [5] |
|  |  | x | bleached (TCF) |  | ~12 | ~250-400 |  |
|  |  | x | bleached + refined |  | ~9 | ~200 |  |
| spruce/ pine | pulp | x | unbleached, unrefined kraft pulp (κ = 42) | pyramidal | ~ 7.0  (45 % RH) | ~ 190  (45 % RH) | [This work] |
|  |  |  |  | hemi-spherical | ~ 8.6  (45 % RH) | ~ 220  (45 % RH) |  |

**S3: Influence of the microfibrillar angle (MFA)**


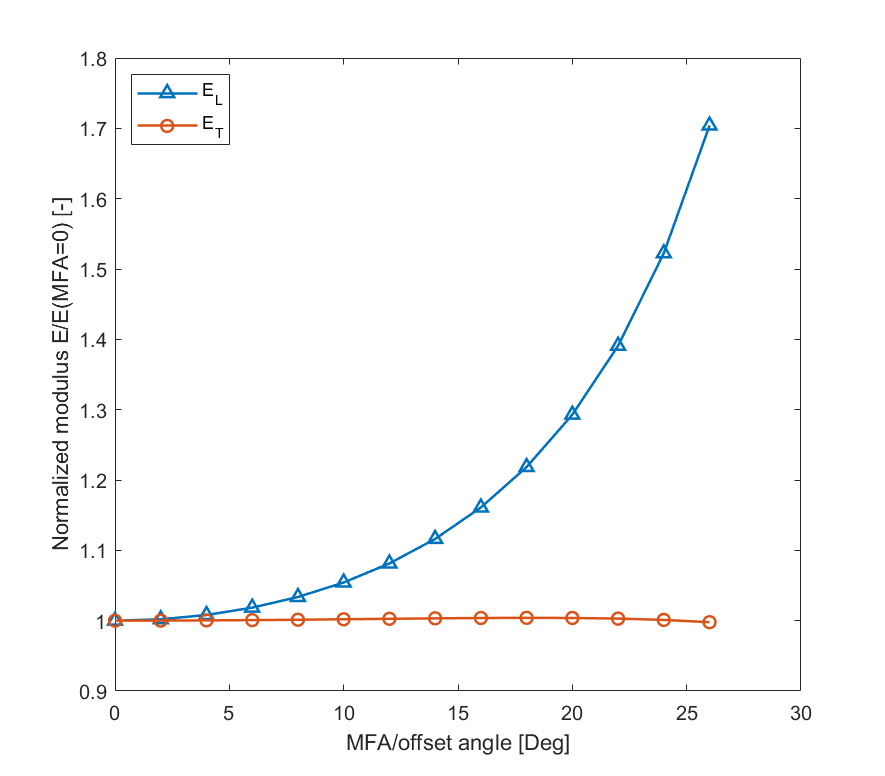


Figure A 4: Estimate of the elastic constants under different assumptions regarding the orientation of the axis of symmetry. The transverse direction is always assumed to be orthogonal to the microfibrils, as the fibrils are known to never point radially outwards from the lumen of the fiber. Calculated using the method of Vlassak et al. [6]

**S4: Comparison of different indenters, different proposed algorithms**


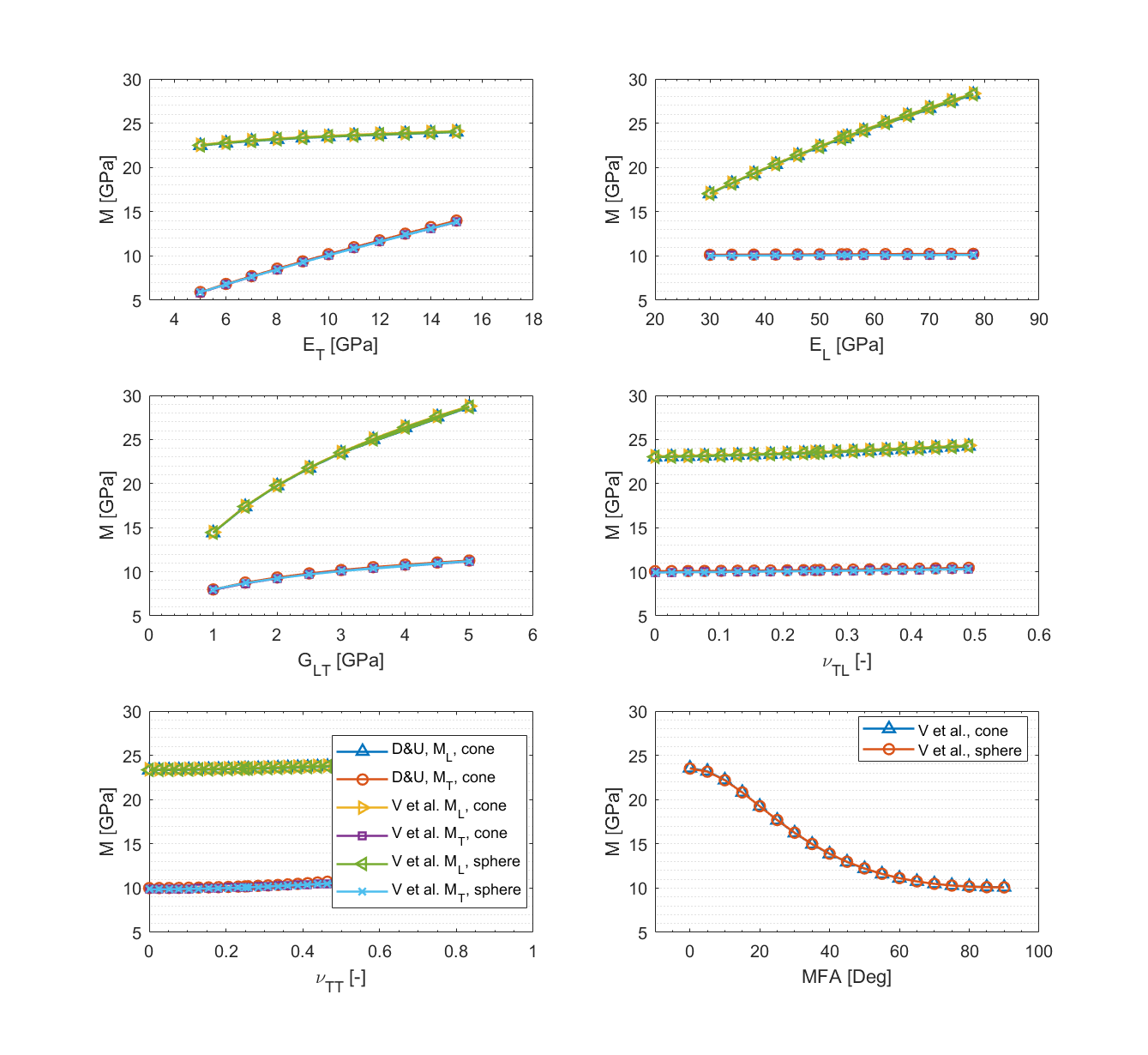


Figure A 5: Variation in output depending on the method of determining the indentation modulus for the direction parallel with and perpendicular to the axis of symmetry. Shown is the theory presented by Vlassak et al. (V et al.) [6] for the case of a cone and a sphere, respectively, as well as the explicit solution of Delafargue and Ulm (D&U) [7]. Although there is a small difference, the results were considered close enough that the Delafargue and Ulm solution, derived for cones, is also valid.

**S5: Control that obtained parameters are a global minimum**

The uniqueness of the obtained moduli pair output by Equation (15) is investigated by performing a grid search over the realistic ranges of longitudinal and transverse moduli. For this purpose, the mean values from the AFM-NI measurements with the hemispherical probe are used as representative experimental data. There is a single cost function minimum. This holds true for all the other combinations used in Figure 8.


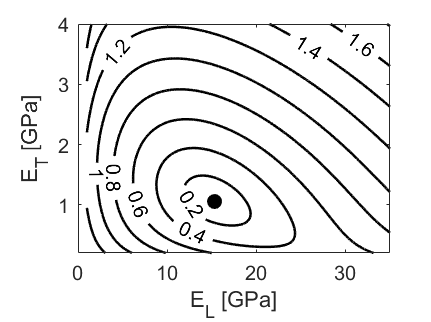


Figure A 6: Grid search over the realistic ranges of moduli using Equation (15) as a cost function and the mean indentation modulus obtained using the hemispherical AFM-NI as experimental input. Labels on contours represent the cost of Equation (15). A unique optimum exists, and it is the same as found using the BFGS-Newton minimization algorithm. This supports the conclusion that the numerical method used to find the longitudinal and transverse fiber modulus is appropriate for this problem.

**Literature**

[1] Wimmer R, Lucas BH, Tsui TY, Oliver WC. Longitudinal hardness and Young’s modulus of spruce tracheid secondary walls using nanoindentation technique. Wood Sci Technol 1997;31:131–41. https://doi.org/10.1007/BF00705928.

[2] Gindl W, Gupta HS, Schöberl T, Lichtenegger HC, Fratzl P. Mechanical properties of spruce wood cell walls by nanoindentation. Appl Phys A Mater Sci Process 2004;79:2069–73. https://doi.org/10.1007/s00339-004-2864-y.

[3] Gindl W, Schöberl T. The significance of the elastic modulus of wood cell walls obtained from nanoindentation measurements. Compos Part A Appl Sci Manuf 2004;35:1345–9. https://doi.org/10.1016/j.compositesa.2004.04.002.

[4] Adusumalli RB, Mook WM, Passas R, Schwaller P, Michler J. Nanoindentation of single pulp fibre cell walls. J Mater Sci 2010;45:2558–63. https://doi.org/10.1007/s10853-010-4226-9.

[5] Adusumalli RB, Passas R, Sreedhar I, Krishnamurthy B, Kombaiah B, Montagne A. Nanoindentation of bleached and refined pulp fibres. Int J Mater Eng Innov 2014;5:138–50.

[6] Vlassak JJ, Ciavarella M, Barber JR, Wang X. The indentation modulus of elastically anisotropic materials for indenters of arbitrary shape. J Mech Phys Solids 2003;51:1701–21. https://doi.org/10.1016/S0022-5096(03)00066-8.

[7] Delafargue A, Ulm FJ. Explicit approximations of the indentation modulus of elastically orthotropic solids for conical indenters. Int J Solids Struct 2004;41:7351–60. https://doi.org/10.1016/j.ijsolstr.2004.06.019.
